# Psychological well-being factors and the likelihood of transitioning from overweight and obesity to normal weight at population level: Evidence from two cohort studies of UK adults

**DOI:** 10.1177/13591053251313589

**Published:** 2025-01-31

**Authors:** I Gusti Ngurah Edi Putra, Michael Daly, Eric Robinson

**Affiliations:** 1 University of Liverpool, UK; 2 Maynooth University, Ireland

**Keywords:** mental health, overweight and obesity reversal, psychological well-being, transitioning into normal weight, weight loss

## Abstract

We examined the prospective associations between psychological well-being related factors (depressive symptoms, life satisfaction, self-efficacy) and transitioning from overweight and obesity to normal body weight (vs persistence of overweight and obesity) and change in body mass index (BMI). We used multiple baselines and follow-ups from the National Child and Development Study (NCDS; 8513 observations) and the British Cohort Study (BCS; 11,113 observations). A proportion (8%–9%) of participants with overweight and obesity (BMI ≥25) at baseline transitioned into normal weight (BMI 18.5–<25) by follow-ups. There was no evidence of better psychological well-being related factors (e.g. lower depressive symptoms) being significantly associated with a transition from overweight and obesity to normal weight or reduced BMI in each cohort and pooled cohort analyses. However, age and gender were associated with transition. At population level, better psychological well-being may not be associated with likelihood of weight loss once obesity is developed in adulthood.

## Introduction

The rising global prevalence of obesity over the past two decades is a significant public health problem (WHO, 2024). In England, more than half of the adult population have lived with overweight or obesity since 1993 and the prevalence of obesity has almost doubled in the last 20 years (Baker, 2023). Living with obesity is prospectively linked to numerous adverse health outcomes (e.g. cardiovascular disease, diabetes, chronic kidney disease) and decreased life expectancy (Putra et al., 2024b; The GBD 2015 Obesity Collaborators, 2017). In line with this, obesity contributes to a substantial economic burden, with recent estimates suggesting that the global economic cost of overweight and obesity is around US$ 2 trillion and is projected to rise to US$ 18 trillion by 2060 (World Obesity Federation, 2022). In the UK, obesity and its associated disease burden contribute to £6.5 billion of NHS annual expenditure (Department of Health and Social Care, 2024).

Given the substantial health and economic consequences of obesity, promoting weight loss in people with overweight and obesity is important due to its observed benefits in reducing risk of developing diabetes and cardiovascular risk factors (Haase et al., 2021). However, transitioning from overweight and obesity to normal weight may be relatively rare once overweight and obesity is developed in adults. Biological adaptations due to weight gain may explain why obesity may be difficult to reverse. Living with obesity is associated with leptin and ghrelin resistance and changes to other gut hormones that may increase appetite and contribute to persistence of current obesity (Blomain et al., 2013; Cui et al., 2017; Lean and Malkova, 2016; Zigman et al., 2016). Nevertheless, some people do transition to a normal weight having lived with overweight and obesity. A large study of healthcare records for English adults showed that approximately 18% of those with overweight successfully transitioned into normal weight (Katsoulis et al., 2021).

Although some studies have examined the transition from overweight or obesity to normal weight in different age groups (Fildes et al., 2015; Häkkänen et al., 2020; Hartono et al., 2021; Hu et al., 2022; Huang and Chen, 2019; Lartey et al., 2020; Lee et al., 2016; Onyimadu et al., 2024; Putra et al., 2024a), there is limited evidence of what factors explain why some adults transition from overweight and obesity to normal weight while others do not. A previous study using data from electronic health records in England indicated that younger age, social deprivation and being part of an ethnic minority were associated with transitioning from normal weight to overweight or obesity in adults (Katsoulis et al., 2021). However, it is unclear whether the opposite characteristics (e.g. less social deprivation) may be associated with transitioning from overweight and obesity to normal weight (vs persistence of overweight and obesity). Better socioeconomic status (SES) may be associated with transitioning from overweight and obesity to normal weight as recent evidence in UK children with overweight indicated that living in lower family income quantiles (vs the highest) is associated with a lower likelihood of transitioning into normal weight (Onyimadu et al., 2024). However, a study in Ghanaian adults showed the opposite findings where individuals from higher SES quintiles had a lower probability of transitioning from overweight to normal weight (Lartey et al., 2020). In earlier studies examining weight change in adults, sociodemographic characteristics, such as gender (Biskup et al., 2021; Finucane et al., 2023; Hejjaji et al., 2022), age (Finucane et al., 2023; Hejjaji et al., 2022) and socioeconomic status (Biskup et al., 2021) are associated with weight loss, presented as reduced body mass index (BMI).

Contemporary theoretical models in health psychology have proposed that the psychological burden of overweight and obesity may in part explain why this condition is hard to reverse. Poor psychological well-being is more common in people living with obesity versus normal weight (Robinson et al., 2020b; Tomiyama, 2014), and this may be associated with subsequent behavioural responses that contribute to maintaining current obesity, such as emotional eating and physical inactivity (Matta et al., 2019; Paans et al., 2018; Tomiyama, 2014). Hypothalamic–pituitary–adrenocortical (HPA) axis activation and increased cortisol levels as a biological response to poor psychological well-being and chronic stress (e.g. weight stigma) may also contribute to persistence of obesity through fat accumulation (Lucassen and Cizza, 2012; Schvey et al., 2014; Tomiyama, 2014). However, studies on what psychological well-being related factors are associated with a transition from overweight and obesity to normal weight are limited with the exception of a recent study in UK children with overweight and obesity that found a range of psychological factors characterised as better mental health and psychosocial well-being (e.g. lower depressive symptoms, greater life satisfaction) were associated with transitioning to normal weight by end of adolescence (Putra et al., 2024a). In line with this, previous studies of adults predominantly enrolled in weight loss interventions found that lower depressive symptoms and positive psychological well-being are linked to better weight loss outcomes (Conradson et al., 2022; Finucane et al., 2023; Ohsiek and Williams, 2011). No studies we are aware of have examined whether psychological well-being related factors (e.g. depressive symptoms, life satisfaction) are associated with the likelihood of transitioning from overweight and obesity to normal weight (vs persistence of overweight and obesity) among the general population, as previous studies included individuals actively engaging in weight loss interventions (Conradson et al., 2022; Finucane et al., 2023; Ohsiek and Williams, 2011).

To address these outstanding questions, we make use of data from two British birth cohorts, namely the National Child and Development Study (NCDS) and the British Cohort Study (BCS), to examine the role of psychological well-being related factors in transitioning from overweight and obesity to normal weight. No studies to date using these cohorts have examined this question (Geoffroy et al., 2014; Mulugeta et al., 2018; Scarpato et al., 2021; White et al., 2012). NCDS and BCS have multi-wave measurements of three psychological well-being related factors: depressive symptoms, life satisfaction and self-efficacy. Given the theorised role of psychological well-being in overweight and obesity maintenance, and well documented relationship these specific psychological well-being factors have with weight gain and weight-related lifestyle behaviour, we hypothesised that better psychological well-being (lower depressive symptoms, higher life satisfaction, higher self-efficacy) would be associated with an increased likelihood of transitioning from overweight and obesity to normal weight.

## Methods

### Data

We used two British cohort datasets, NCDS (Power and Elliott, 2005) and BCS (Elliott and Shepherd, 2006). These cohorts recruited individuals born in three UK countries: England, Scotland and Wales in a single week 12 years apart, in 1958 and 1970, respectively. In both cohorts, follow-up interviews were carried out from ages 7 (1965) to 62 (2020–2024) in NCDS and from ages 5 (1975) to 51 (2021–2024) in BCS. As we examined a transition or movement from overweight and obesity to normal weight category in young and middle-aged adulthood, we used data collected at ages 23, 33, 42 and 50 for NCDS and 26, 30, 34, 42 and 46 for BCS in which information on psychological well-being related factors and BMI was consistently available across waves of interest (e.g. as in Putra et al. (2024c)). Multicentre Research Ethics Committee approved NCDS and BCS. All the participants provided informed consent.

### Transitioning from overweight and obesity to normal weight

We used harmonised BMI data produced by the Centre for Longitudinal Study, UCL (Bann et al., 2017; CLOSER, 2022; Johnson et al., 2015). BMI across waves in both cohorts was based on available self-reported or objectively measured weight and height (self-reported data were available in most waves: ages 22, 42 and 50 in NCDS and ages 26, 30, 34, 42 and 46 in BCS; objectively measured data collected by nurses were available at age 33 in NCDS and age 46 in BCS). When both self-reported and objectively measured data were available to calculate BMI (age 46 in BCS, both objective and self-reported BMI were strongly correlated, *r* = 0.88), objectively measured BMI were preliminary computed, with self-reported BMI used as substitutes. Following a previous study (Putra et al., 2024a), we defined a transition from overweight and obesity to normal weight as a change between two survey waves from living with overweight and obesity (BMI ≥25) at baseline to normal body weight category (BMI 18.5–<25) at follow-up (see ‘Data Analysis’). We did not assess whether participants previously had lived with normal body weight before having overweight and obesity at baseline. We excluded cohort members with normal weight at baseline. We also removed a small proportion of cohort members with underweight (BMI <18.5) at baseline and follow-up as poorer psychological well-being is predictive of underweight (Geoffroy et al., 2014).

### Psychological well-being related factors

We followed a previous approach (Putra et al., 2024c) to examine depressive symptoms, life satisfaction and self-efficacy as psychological well-being of interests given these factors were consistently collected across waves in both cohorts. The following describes how each measure was quantified following previous studies. The summary or total scores in different matrices were transformed into *z*-scores to make them comparable.

**Figure 1.**
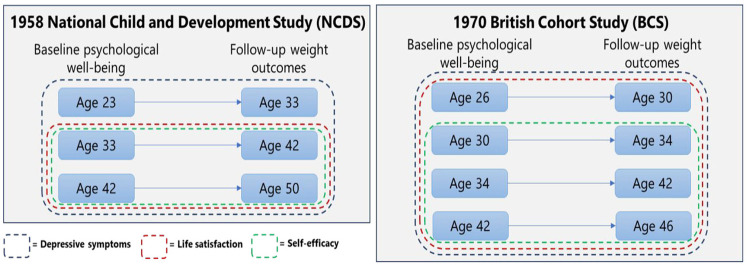
Pooled analyses of multiple pairs of baseline and follow-up in each cohort (See pre-registered analysis protocol: https://doi.org/10.17605/OSF.IO/2QEPT). Only observations with overweight and obesity at baseline were included.

A validated nine-item Malaise Inventory version (McGee et al., 1986; Rodgers et al., 1999) was used to quantify depressive symptoms (e.g. as in Arias-de la Torre et al. (2021)). Cohort members responded to dichotomous questions (yes, no) on whether they experienced negative emotions (e.g. ‘*Do you often feel miserable or depressed?*’). We computed a total score on a scale of 0–9 with a higher score indicative of higher depressive symptoms. Depressive symptoms were measured from young ages, and therefore, our analysis for this psychological measure used all the specified waves in NCDS and BCS (see Figure 1).

A response to a single 10-point Likert-scale item, ‘*How dissatisfied or satisfied you are about the way your life has turned out so far?*’ was used to evaluate life satisfaction (e.g. as in Flèche et al. (2021); Hatch et al. (2010)). Better life satisfaction was indicated by a higher score ranging from 0 to 10. The analysis for life satisfaction used datasets from when it was collected (from ages 33 and 26 in NCDS and BCS, respectively) (see Figure 1).

Three items (e.g. ‘*Usually I can run my life more or less as I want to*’) with dichotomous responses were used to determine self-efficacy (e.g. as in Hatch et al. (2010)). A total score on a scale of 0–3 was generated by totalling all the responses with higher self-efficacy indicated by a higher score. Given this measure was not collected from earlier waves, the analysis for self-efficacy was limited to datasets from ages 33 and 30 in NCDS and BCS, respectively (see Figure 1).

### Sociodemographic covariates

We controlled for baseline sociodemographic characteristics and SES measures available in the datasets (e.g. as in Putra et al. (2024c)). This included age at data collection, sex (male, female), ethnicity (defined as non-White and White due to a small proportion of participants who were ethnic minorities) and current legal marital status (single or never married, married and an additional group of others: divorced, separated, widowed). Four measures of SES (father’s and cohort members’ occupational groups, and cohort members’ educational level and housing tenure status) were selected. We followed the classification used in a previous study (Daly and Egan, 2017), categorising occupation into five groups: (1) professional, (2) intermediate (managerial and technical), (3) skilled, (4) partly skilled and (5) unskilled, and a separate group was created for unemployment and occupations that did not fit into any of the specified groups. Educational level was classified based on National Vocational Qualification (NVQ) groups, ranging from NVQ-1 for qualifications equivalent to vocational or second-level education to NVQ-5 for postgraduate qualifications (e.g. as in Daly and Egan (2017)). Following a previous approach (Geoffroy et al., 2014), a binary classification of owner-occupier and others was used to present housing tenure status.

### Data analysis

We pre-registered our analysis protocol (https://doi.org/10.17605/OSF.IO/2QEPT). We examined the associations between psychological well-being related factors and the transition from overweight and obesity to normal weight controlling for sociodemographic covariates and baseline BMI. We combined pairs of baseline and follow-up and conducted a pooled analysis separately in each cohort (e.g. as in Geoffroy et al. (2014); Putra et al. (2024c)). Age- or wave-varying psychological well-being related factors at ages 23, 33 and 42 (baseline) were fitted to predict a transition from overweight and obesity to normal weight (vs persistence of overweight and obesity) at ages 33, 42 and 50 (follow-up), respectively, in NCDS, and we used the same approach for BCS (see Figure 1). We used different analytical sample sizes when analysing psychological well-being related factors as these factors were not collected from the same wave (see ‘Psychological well-being related factors’ and Figure 1). To account for correlated observations and hierarchical data structure with observations (or waves) nested within individuals, we used panel data analysis in STATA (‘xtlogit’ command with ‘vce (cluster participant_ID)’ option).

Our analysis omitted observations with missing weight status. We included 8513 and 11,113 observations with overweight or obesity at baseline nested within 5260 and 5530 individuals in NCDS and BCS, respectively. We used multiple imputations by chained equations (MICE; Azur et al., 2011; White et al., 2011; ‘mi impute chained’ command in STATA) to impute missing information on other variables. In addition to including main variables for the analysis, some auxiliary variables (cohort members’ birth weight, breastfeeding habits, maternal smoking status and mother’s age at the time of cohort member’s birth) available across cohorts were selected to improve the imputation model following previous studies (Khanolkar and Patalay, 2021; Putra et al., 2024c). We set statistical significance at *p*-value <0.05 for the main analysis above (i.e. investigating the categorical outcome of the transition to normal weight). As we conducted multiple testing for additional analyses (see below), this may lead to increased rates of false positives or Type 1 errors. Therefore, we controlled false discovery rates at 5% using the Benjamini-Hochberg (BH) method (Benjamini and Hochberg, 1995). This method provides an adjusted threshold for each *p*-value, and statistically significant associations are determined based on whether the *p*-value is smaller than the corresponding threshold (see Supplemental Materials).

We also examined the outcome in models as a continuous variable, presented as residualised BMI change scores. We computed residualised change values, independent of baseline BMI, by fitting a regression model of follow-up BMI predicted by baseline BMI (e.g. as in Deforche et al. (2015); Putra et al. (2024c)). For this analysis, we used the same analytical sample sizes as the main analysis to evaluate the associations between psychological well-being related factors and residualised BMI change scores (‘xtreg’ command with ‘vce (cluster participant_ID)’ option) separately in each cohort. For analysis with residualised BMI change scores as the outcome, we did not control for baseline BMI.

While the main and additional analyses above were specific using a shorter follow-up period (ranging from 4 to 10 years with 6–7 years on average, see Figure 1), we replicated the analyses to assess associations between psychological well-being factors and the transition from overweight and obesity to normal weight using partial analytical sample sizes with longer follow-up in each cohort. We selected ages 33 in NCDS and 30 in BCS as baseline as these were the first waves where all three psychological well-being related factors were collected, and then used ages 50 in NCDS and 46 in BCS as follow-up (16–17 years of follow-up; e.g. as in Putra et al. (2024c)).

We examined the associations using a bigger analytical sample size. We conducted pooled analyses for shorter and longer follow-ups combining analytical sample sizes from both cohorts. In addition, we aimed to understand whether associations differed by cohort. Interaction terms between the cohort (BCS vs NCDS) and factors of psychological well-being were included in the separate regression models in predicting the outcomes (transitioning to normal weight and BMI change).

## Results

Characteristics of observations across the cohort studies were comparable with slightly higher proportions of males (57%–59%) versus females (41%–43%), mostly White participants (99%), and more than half were married (55%–70%; Table 1). Based on SES, 68% were in intermediated and skilled occupation groups. Around one-third (30%–33%) completed a qualification equivalent to NVQ level 2 in NCDS, either NVQ level 2 or level 4 in BCS. 70% of the cohort members were home owner-occupiers. Across studies, 8%–9% of overweight and obesity cases at baseline transitioned into normal weight at follow-up.

**Table 1.** Participants’ observations with overweight and obesity across the study baseline, pooled by cohort.

Variables	NCDS		BCS	
	*n* = 8513^a^	%	*n* = 11,113^a^	%
Sex				
Male	5038	59.18	6314	56.82
Female	3475	40.82	4799	43.18
Age (years)				
23	1452	17.06	*N/A*	*N/A*
26	*N/A*	*N/A*	1525	13.72
30	*N/A*	*N/A*	3197	28.77
33	3559	41.81	*N/A*	*N/A*
34	*N/A*	*N/A*	3124	28.11
42	3502	41.14	3267	29.40
Ethnicity				
White	8462	99.40	10,990	98.89
Non-White	33	0.39	68	0.61
*Missing*	*18*	*0.21*	*55*	*0.49*
Marital status				
Single	1562	18.35	4000	35.99
Married	5979	70.23	6155	55.39
Others	878	10.31	931	8.38
*Missing*	*94*	*1.10*	*27*	*0.24*
Father’s occupation				
Professional	281	3.30	478	4.30
Intermediate	1291	15.17	2370	21.33
Skilled	3938	46.16	5079	45.70
Partly skilled	1103	12.96	1252	11.27
Unskilled	643	7.55	590	5.31
Others	302	3.55	274	2.47
*Missing*	*955*	*11.22*	*1070*	*9.63*
Cohort member’s occupation				
Professional	355	4.17	516	4.64
Intermediate	2307	27.10	3605	32.44
Skilled	3528	41.44	3962	35.65
Partly skilled	1122	13.18	1081	9.73
Unskilled	311	3.65	221	1.99
Others	890	10.45	1728	15.55
Cohort member’s education				
No qualification	1051	12.35	1183	10.65
NVQ level 1	1090	12.80	950	8.55
NVQ level 2	2806	32.96	3358	30.22
NVQ level 3	1249	14.67	1754	15.78
NVQ level 4	1077	12.65	3346	30.11
NVQ level 5	703	8.26	519	4.67
*Missing*	*537*	*6.31*	*3*	*0.03*
Housing tenure status				
Owner-occupier	5915	69.48	7812	70.30
Other	2281	26.79	3246	29.21
*Missing*	*317*	*3.72*	*55*	*0.49*
BMI category at baseline				
Overweight	6413	75.33	7796	70.15
Obesity	2100	24.67	3317	29.85
Transitioning from overweight and obesity to normal weight				
Yes	731	8.59	883	7.95
No (or persistence)	7782	91.41	10,230	92.05

%: percentage; N/A: not applicable due to no data collection at those ages or waves (see below); NVQ: National Vocational Qualification.

a Number of eligible observations nested within individuals. Observations at ages 23, 33 and 42 in NCDS, and ages 26, 30, 34 and 42 in BCS were excluded if participants had normal weight (BMI 18.5–<25) at baseline and underweight (BMI <18.5) at baseline and follow-up.

Table 2 presents associations between psychological well-being related factors (depressive symptoms, life satisfaction, self-efficacy) and the transition from overweight and obesity to normal weight (vs persistence of overweight and obesity), while Table 3 presents the associations when BMI change was examined as the outcome. Tables 4 and 5 replicated the analysis presented in Tables 2 and 3, respectively, using partial analytical sample size with a longer follow-up period. Other findings from additional analyses are presented in Supplemental Materials.

**Table 2.** Psychological well-being related factors and transitioning from overweight and obesity to normal weight.

Psychological well-being related factors	Transitioning from overweight and obesity to normal weight vs persistence of overweight and obesity							
	NCDS				BCS			
	*n*	OR	95% CI	*p*-Value	*n*	OR	95% CI	*p*-Value
Depressive symptoms	8513	1.09	0.98, 1.21	0.105	11,113	0.99	0.92, 1.08	0.862
Life satisfaction	7061	1.01	0.90, 1.15	0.821	11,113	1.02	0.95, 1.11	0.565
Self-efficacy	7061	0.95	0.85, 1.07	0.423	9588	1.07	0.97, 1.19	0.176

Depressive symptoms, life satisfaction and self-efficacy were transformed into *z*-scores. Associations between psychological well-being related factors and the outcome were fitted in separate regression models, controlling for sociodemographic covariates and baseline BMI.

*n*: number of observations; OR: odds ratio; CI: confidence intervals.

**Table 3.** Psychological well-being related factors and BMI change.

Psychological well-being related factors	Changes in BMI							
	NCDS				BCS			
	*n*	β	95% CI	*p*-Value	*n*	β	95% CI	*p*-Value
Depressive symptoms	8513	−0.00	−0.08, 0.07	0.919	11,113	0.04	−0.02, 0.10	0.205
Life satisfaction	7061	−0.06	−0.14, 0.02	0.118	11,113	−0.03	−0.09, 0.03	0.357
Self-efficacy	7061	−0.04	−0.12, 0.05	0.404	9588	−0.04	−0.11, 0.02	0.201

*n*: number of observations; β: regression coefficient; CI: confidence intervals.

Depressive symptoms, life satisfaction and self-efficacy were transformed into *z*-scores. Associations between psychological well-being related factors and the outcome were fitted in separate regression models, controlling for sociodemographic covariates.

**Table 4.** Psychological well-being related factors and transitioning from overweight and obesity to normal weight (using partial analytical sample sizes with a longer follow-up period).

Psychological well-being related factors	Transitioning from overweight and obesity to normal weight vs persistence of overweight and obesity							
	NCDS				BCS			
	Baseline: age 33, follow-up: age 50				Baseline: age 30, follow-up: age 46			
	*n*	OR	95% CI	*p*-Value	*n*	OR	95% CI	*p*-Value
Depressive symptoms	2600	0.98	0.82, 1.17	0.825	2316	0.83	0.66, 1.04	0.100
Life satisfaction	2600	1.01	0.84, 1.21	0.922	2316	1.12	0.90, 1.39	0.327
Self-efficacy	2600	0.97	0.81, 1.16	0.718	2316	1.11	0.88, 1.38	0.384

Depressive symptoms, life satisfaction and self-efficacy were transformed into *z*-scores. Associations between psychological well-being related factors and the outcome were fitted in separate regression models, controlling for sociodemographic covariates and baseline BMI. However, ethnicity was not controlled due to a very small number of participants who were non-White.

*n*: number of observations; OR: odds ratio; CI: confidence intervals

**Table 5.** Psychological well-being related factors and BMI change (using partial analytical sample sizes with a longer follow-up period).

Psychological well-being related factors	Changes in BMI							
	NCDS				BCS			
	Baseline: age 33, follow-up: age 50				Baseline: age 30, follow-up: age 46			
	*n*	β	95% CI	*p*-Value	*n*	β	95% CI	*p*-Value
Depressive symptoms	2600	0.12	−0.03, 0.27	0.106	2316	0.17	−0.01, 0.35	0.067
Life satisfaction	2600	−0.00	−0.16, 0.15	0.965	2316	−0.09	−0.28, 0.09	0.317
Self-efficacy	2600	−0.09	−0.24, 0.06	0.259	2316	0.03	−0.16, 0.21	0.779

Depressive symptoms, life satisfaction and self-efficacy were transformed into z-scores. Associations between psychological well-being related factors and the outcome were fitted in separate regression models, controlling for sociodemographic. However, ethnicity was not controlled due to a very small number of participants who were non-White.

*n*: number of observations; β: regression coefficient; CI: confidence intervals.

We found none of the psychological well-being related factors were prospectively associated with the transition to normal weight across both cohorts (Table 2). Findings were consistent when BMI change scores were examined as the outcome (Table 3). In additional analyses limited to observations with a longer follow-up period (16–17 years), psychological well-being related factors were consistently not associated with the transition from overweight and obesity to normal weight (Table 4) or BMI change (Table 5).

Increasing the analytical power by combining analytical sample sizes from both cohorts (a pooled cohort analyses) yielded the same findings, as psychological well-being related factors were not associated with either the transition from overweight and obesity to normal weight or changes in BMI (Table S1). No evidence for statistically significant differences in the effect sizes of the associations between NCDS and BCS was observed in the cohort-pooled analysis (Table S2). Similarly, a pooled analysis for partial analytical sample sizes with a longer follow-up period from both cohorts showed no evidence for significant associations between psychological well-being related factors and the outcomes (Table S3) and differences in the effect sizes between cohorts (Table S4).

Having confirmed no evidence for psychological well-being related factors predicting the transition from overweight and obesity to normal weight, we examined whether sociodemographic characteristics were linked to the transition (Table S5). We found that a higher baseline age up to 34 years (vs age 23) was associated with increased odds of transitioning to normal weight, with a statistically significant association observed at age 33 (vs age 23). However, participants aged 42 (vs age 23) at baseline were associated with a lower likelihood of transitioning into normal weight. Furthermore, females (vs males) were more likely to transition to normal weight at follow-up.

Consistent with previous research (Katsoulis et al., 2021), we observed more transitions from overweight to normal weight than from obesity at baseline (11%; 1540 out of 14,209 observations with overweight vs 1%; 74 out of 5417 observations with obesity). With a high baseline proportion of obesity (28%; 5417 out of 19,626 observations), but a lower proportion of transitions to normal weight from this BMI category (1%), psychological well-being related factors may have limited predictive ability for transition to normal weight when observations with obesity are included in the analysis. Therefore, we conducted a non-preregistered pooled analysis using full analytical sample sizes due to its greater analytical power to explore whether psychological well-being related factors are associated with a transition from overweight alone to normal weight (as opposed to combined overweight and obesity at baseline). We found that in participants with overweight (BMI 25–<30) at baseline (Table S6), the findings were consistent with the main analysis (the transition from overweight and obesity to normal weight, Table 1) as no psychological well-being related factors were associated with a transition from overweight to normal weight.

## Discussion

We examined the extent to which three psychological well-being related factors (depressive symptoms, life satisfaction, self-efficacy) were associated with the likelihood of transitioning from overweight and obesity (BMI ≥25) into the normal weight category (BMI 18.5–<25) in two large longitudinal cohort studies of UK adults aged between 23 and 50 years. Transitioning into the normal weight BMI category was consistently observed but relatively rare (8%–9%). There was no convincing evidence that the examined psychological well-being related factors were related to the likelihood of transitioning from overweight and obesity into the normal weight category. However, some demographic factors were associated with transitioning from the overweight/obesity to normal weight BMI category, whereby females and adults younger than 42 years were more likely to transition to a BMI in the normal weight category.

Consistent with previous research (Katsoulis et al., 2021; Lartey et al., 2020), we found that a small but significant minority of participants with overweight and obesity lost significant weight and transitioned to the normal weight BMI category across study follow-ups. Based on contemporary models in health psychology which suggest that impaired psychological well-being may explain why overweight and obesity can be difficult to reverse (Hunger et al., 2015; Robinson et al., 2020a; Tomiyama, 2014), we predicted that better psychological well-being would be associated with an increased likelihood of transitioning from overweight and obesity to normal weight. In line with this, recent research examining transitioning out of overweight and obesity and into normal weight among UK children in a large cohort study, found that better baseline psychological well-being (e.g. lower depression) at age 11, but not age 14 was found to be associated with increased likelihood of transition by age 17 (Putra et al., 2024a). It is therefore striking that we found no evidence that psychological well-being related factors were associated with transitioning to normal weight from overweight and obesity across multiple cohorts of UK adults. This may be due to differences in developmental adaptability as adulthood is characterised by slower metabolic rates compared to childhood and adolescence (Pontzer et al., 2021), and therefore, transitioning from overweight and obesity to normal weight may be more challenging. In addition, better psychological well-being often coincidences with supportive environments in younger individuals (Uzun et al., 2023) and successful weight loss in this age group could be motivated by social influence (Kulik et al., 2016; LaRose et al., 2013). Meanwhile, weight loss in adults may be more motivated by health concerns (LaRose et al., 2013), and this may not often necessarily precede or coincidence with better psychological well-being.

A number of studies have shown that psychological well-being related factors such as depression (Blaine, 2008; Luppino et al., 2010; Mannan et al., 2016; Shell et al., 2024) and lower life satisfaction (Korkeila et al., 1998) are associated with weight change. In the present study, we found that better psychological well-being related factors did not predict a prospective decrease in BMI in participants with overweight and obesity at baseline across individual and pooled cohort analyses. Therefore, although psychological well-being related factors may play a role in explaining the development of overweight and obesity, once obesity is developed and has become a chronic and long-term condition, psychological well-being may not contribute to further weight gain or likelihood of significant weight loss. Some recent work in part supports the proposition that psychological well-being in adulthood may predominantly only relate to development of obesity but not further trajectories of weight. Using the same two cohorts as in the present research, there was evidence of psychological well-being related factors predicting weight gain among participants without obesity, but not among participants with obesity (Putra et al., 2024c). Findings from a study among Dutch participants also supported this proposition (Cloostermans et al., 2015). However, more direct testing of this proposition is now warranted.

It is important to note that the present research examined participants from the general population, unlike past research which has examined predictors of weight loss among smaller groups of participants self-enrolled in structured weight loss programmes or undergoing bariatric surgery. There is some evidence linking better mental health to improved weight loss outcomes among adults engaged in lifestyle programmes for weight loss and bariatric surgery in a small number of studies (Finucane et al., 2023; Varkevisser et al., 2019). However, there is a lack of research on psychological predictors of weight loss in such interventions and only a small number of studies are available to date (Varkevisser et al., 2019), therefore further research in these populations and the general population would now be valuable.

Because there are significant health benefits of even modest weight loss (Finucane et al., 2023), developing a better understanding of modifiable factors that increase likelihood of transitioning from overweight and obesity to normal weight is important. Due to availability of data in the cohort studies used, we were only able to examine a limited number of psychological well-being related factors. Additional research examining a wider range of relevant psychological factors would now be valuable. For example, at present, we are aware of no research directly examining how other theoretically relevant constructs hypothesised to explain maintenance of overweight and obesity, such as internalised weight stigma (Bidstrup et al., 2022), relate to likelihood of significant long-term weight loss in the general population. A related psychological factor that may be of relevance to the transition from overweight and obesity to normal weight in adulthood is experiencing weight-based discrimination, as in one US cohort study experiencing weight-based discrimination was associated with increased odds of remaining in the obesity BMI category at 4-year follow-up (Sutin and Terracciano, 2013). However, in a similar UK cohort study, odds of remaining in the obesity BMI category over time did not differ according to experiences of weight discrimination (Jackson et al., 2014).

There are limitations to the present research. We only examined the relatively small number of psychological well-being related factors. Future studies will benefit from testing a range of other psychological well-being related factors using validated measuring instruments (e.g. enjoyment of life, purpose in life, positive affect, etc.; Putra et al., 2023). Study results were largely based on self-reported weight and height to calculate BMI. Although self-reported BMI is strongly correlated with objectively measured BMI and is widely considered to be a reasonable proxy (Davies et al., 2020). Findings may be specific to the populations sampled and time period when sampled. The results may therefore not be generalisable to older adults, predominantly non-White populations and cohorts conducted more recently (last follow-ups examined in the present study were in 2016). Analyses were also unable to account for the likely reasons for weight loss among the 8%–9% of cohort members that transitioned into normal weight category. We presume that due to the age of participants sampled, unintentional weight loss due to illness would be rare and any significant weight loss would therefore likely have to be intentional. However, it would be preferable for future research to use studies which measure weight loss intentions, as it is feasible that psychological well-being could be directionally associated with both intentional (positively) and unintentional health-related (negatively) weight loss in general population samples.

Strengths of the present research include the use of multi-wave cohort studies with analyses examining both short-term and long-term follow-ups, as well as replication of results across two distinct cohorts. As transitioning from overweight and obesity BMI to normal weight BMI is a relatively rare event (<10% in the present research), a further strength of the present research was the very large overall sample sizes used resulted in statistical models being able to predict a reasonably large number of cases (1300–1614) of transitions from overweight and obesity to normal weight in pooled analyses of short-term follow-up. Importantly, secondary analyses examining BMI as a continuous outcome were not limited in sample size for cases. Therefore, the lack of evidence for psychological well-being related factors being associated with weight-related outcomes when examined as the transition into normal weight (relatively rare categorical outcome) or change in BMI (continuous outcome) is unlikely to be explained by low statistical power.

### Conclusion

Among UK adults in the general population, less than 10% transitioned from overweight and obesity to normal body weight. Better psychological well-being related factors did not prospectively predict transition into normal weight in individuals with overweight and obesity and were not associated with weight loss once obesity is developed in the general adult population. Findings indicate that psychological well-being may have a limited role in explaining weight loss in the general population.

## Supplemental Material

sj-docx-1-hpq-10.1177_13591053251313589 – Supplemental material for Psychological well-being factors and the likelihood of transitioning from overweight and obesity to normal weight at population level: Evidence from two cohort studies of UK adults Supplemental material, sj-docx-1-hpq-10.1177_13591053251313589 for Psychological well-being factors and the likelihood of transitioning from overweight and obesity to normal weight at population level: Evidence from two cohort studies of UK adults by I Gusti Ngurah Edi Putra, Michael Daly and Eric Robinson in Journal of Health Psychology
