# Supplementary material for: Psychological well-being factors and the likelihood of transitioning from overweight and obesity to normal weight at population level: Evidence from two cohort studies of UK adults
Source: J Health Psychol. 2025 Jan 31;30(13):3770–85. doi: 10.1177/13591053251313589 (PMC12618731; doi:10.1177/13591053251313589)
Supplement: sj-docx-1-hpq-10.1177_13591053251313589 – Supplemental material for Psychological well-being factors and the likelihood of transitioning from overweight and obesity to normal weight at population level: Evidence from two cohort studies of UK adults [file sj-docx-1-hpq-10.1177_13591053251313589.docx]

**Supplementary materials**

We conducted additional analyses to examine the consistency of the findings from the main analysis. The purposes of the supplementary tables presented are as follows:

- Table S1: To confirm the findings from Tables 2 and 3 (in the main text) by combining the analytical sample sizes from both cohorts (NCDS and BCS).
- Table S2: To explore whether the associations between psychological well-being factors and the outcomes in cohort-pooled analyses (Table S1) differed between the NCDS and BCS cohorts.
- Table S3: To confirm the findings from Tables 4 and 5 (in the main text) by combining the partial analytical sample size with a longer follow-up from both cohorts (NCDS and BCS).
- Table S4: To explore whether the associations between psychological well-being factors and the outcomes in cohort-pooled analyses with a longer follow-up period (Table S3) differed between the NCDS and BCS cohorts.
- Table S5: To examine the associations between sociodemographic characteristics and transitioning from overweight and obesity to normal weight, as we found null associations for psychological well-being-related factors.
- Table S6: To examine the associations between psychological well-being-related factors and transitioning from overweight to normal weight (excluding those living with obesity at baseline), as we found more transitions from overweight to normal body weight.
- Table S7: To control for the false discovery rate using the Benjamini-Hochberg method.

**Table S1.** Psychological well-being related factors and the outcomes (using pooled analytical sample sizes from both cohorts)

| **Psychological well-being related factors** | **Transitioning from overweight and obesity to normal weight vs. persistence of overweight and obesity** | | | | **Changes in BMI** | | | |
| --- | --- | --- | --- | --- | --- | --- | --- | --- |
|  | **n** | **OR** | **95% CI** | **p-value** | **n** | **β** | **95% CI** | **p-value** |
| Depressive symptoms | 19,626 | 1.03 | 0.97, 1.10 | 0.278 | 19,626 | 0.02 | -0.03, 0.07 | 0.418 |
| Life satisfaction | 18,174 | 1.02 | 0.96, 1.09 | 0.558 | 18,174 | -0.03 | -0.09, 0.01 | 0.105 |
| Self-efficacy | 16,649 | 1.02 | 0.95, 1.09 | 0.672 | 16,649 | -0.04 | -0.09, 0.01 | 0.157 |

*n=number of observations; OR = odds ratio; β = regression coefficient; CI = confidence intervals*

*Depressive symptoms, life satisfaction, and self-efficacy were transformed into z-scores.*

*Associations between psychological well-being related factors and the outcomes were fitted in separate regression models, controlling for sociodemographic covariates, baseline BMI (for categorical outcome), and the cohort.*

**Table S2.** Interactions between psychological well-being related factors and the study cohort in predicting the outcomes (using pooled analytical sample sizes from both cohorts)

| **Psychological well-being related factors * Cohort (BCS vs. NCDS)** | **Transitioning from overweight and obesity to normal weight vs. persistence of overweight and obesity** | | | | **Changes in BMI** | | | |
| --- | --- | --- | --- | --- | --- | --- | --- | --- |
|  | **n** | **OR** | **95% CI** | **p-value** | **n** | **β** | **95% CI** | **p-value** |
| Depressive symptoms * BCS | 19,626 | 0.92 | 0.82, 1.03 | 0.153 | 19,626 | -0.01 | -0.08, 0.06 | 0.832 |
| Life satisfaction * BCS | 18,174 | 1.04 | 0.91, 1.18 | 0.598 | 18,174 | 0.03 | -0.07, 0.12 | 0.548 |
| Self-efficacy * BCS | 16,649 | 1.11 | 0.97, 1.27 | 0.138 | 16,649 | -0.01 | -0.11, 0.09 | 0.891 |

*n=number of observations; OR = odds ratio; β = regression coefficient; CI = confidence intervals*

*Depressive symptoms, life satisfaction, and self-efficacy were transformed into z-scores.*

*Interactions between psychological well-being related factors and the cohort in predicting the outcomes were fitted in separate regression models, controlling for sociodemographic covariates, baseline BMI (for categorical outcome), corresponding psychological well-being related measure, and the cohort.*

**Table S3.** Psychological well-being related factors and the outcomes (using pooled partial analytical sample sizes with a longer follow-up period from both cohorts)

| **Psychological well-being related factors** | **NCDS: Baseline: age 33, follow-up: age 50**  **BCS: Baseline: age 30, follow-up: age 46** | | | | | | | |
| --- | --- | --- | --- | --- | --- | --- | --- | --- |
|  | **Transitioning from overweight and obesity to normal weight vs. persistence of overweight and obesity** | | | | **Changes in BMI** | | | |
|  | **n** | **OR** | **95% CI** | **p-value** | **n** | **β** | **95% CI** | **p-value** |
| Depressive symptoms | 4,916 | 0.92 | 0.80, 1.06 | 0.243 | 4,916 | 0.13 | 0.01, 0.25 | 0.028 |
| Life satisfaction | 4,916 | 1.06 | 0.92, 1.22 | 0.440 | 4,916 | -0.05 | -0.17, 0.07 | 0.429 |
| Self-efficacy | 4,916 | 1.02 | 0.89, 1.17 | 0.809 | 4,916 | -0.03 | -0.15, 0.09 | 0.618 |

*n=number of observations; OR = odds ratio; β = regression coefficient; CI = confidence intervals*

*Depressive symptoms, life satisfaction, and self-efficacy were transformed into z-scores.*

*Associations between psychological well-being related factors and the outcomes were fitted in separate regression models, controlling for sociodemographic covariates, baseline BMI (for categorical outcome), and the cohort. However, ethnicity was not controlled due to a very small number of participants who were non-White.*

**Table S4.** Interactions between psychological well-being related factors and the study cohort in predicting the outcomes (using pooled partial analytical sample sizes with a longer follow-up period from both cohorts)

| **Psychological well-being related factors * Cohort (BCS vs. NCDS)** | **NCDS: Baseline: age 33, follow-up: age 50**  **BCS: Baseline: age 30, follow-up: age 46** | | | | | | | |
| --- | --- | --- | --- | --- | --- | --- | --- | --- |
|  | **Transitioning from overweight and obesity to normal weight vs. persistence of overweight and obesity** | | | | **Changes in BMI** | | | |
|  | **n** | **OR** | **95% CI** | **p-value** | **n** | **β** | **95% CI** | **p-value** |
| Depressive symptoms * BCS | 4,916 | 0.82 | 0.62, 1.08 | 0.156 | 4,916 | 0.22 | -0.01, 0.44 | 0.062 |
| Life satisfaction * BCS | 4,916 | 1.10 | 0.84, 1.45 | 0.494 | 4,916 | -0.14 | -0.37, 0.09 | 0.238 |
| Self-efficacy * BCS | 4,916 | 1.15 | 0.87, 1.51 | 0.339 | 4,916 | 0.02 | -0.21, 0.25 | 0.870 |

*n=number of observations; OR = odds ratio; β = regression coefficient; CI = confidence intervals*

*Depressive symptoms, life satisfaction, and self-efficacy were transformed into z-scores.*

*Interactions between psychological well-being related factors and the cohort in predicting the outcomes were fitted in separate regression models, controlling for sociodemographic covariates, baseline BMI (for categorical outcome), corresponding psychological well-being related measure, and the cohort. However, ethnicity was not controlled due to a very small number of participants who were non-White.*

**Table S5.** Sociodemographic characteristics and transitioning from overweight and obesity to normal weight (using pooled analytical sample sizes from both cohorts)

| **Sociodemographic characteristics** | **Transitioning from overweight and obesity to normal weight vs. persistence of overweight and obesity**  **(n = 19,626)** | | | |
| --- | --- | --- | --- | --- |
|  | **OR** | **95% CI** | **p-value** | **Overall p-value** |
| Age *(ref: 23)*  26  30  33  34  42 | 1.31  1.26  1.61  1.35  0.62 | 0.91, 1.87  0.90, 1.78  1.28, 2.04  0.95, 1.91  0.47, 0.80 | 0.141  0.183  <0.001  0.091  <0.001 | <0.001* |
| Sex (*ref: Male)*  Female | 1.78 | 1.56, 2.03 | <0.001 | <0.001* |
| Ethnicity *(ref: Non-White)*  White | 1.58 | 0.58, 4.26 | 0.371 | 0.371 |
| Marital status *(ref: Married)*  Single  Others | 0.96  0.94 | 0.83, 1.11  0.76, 1.17 | 0.562  0.607 | 0.779 |
| Father’s occupation *(ref: Professional)*  Intermediate  Skilled  Partly skilled  Unskilled  Others | 0.97  0.91  0.91  0.70  0.82 | 0.71, 1.33  0.67, 1.24  0.64, 1.29  0.47, 1.05  0.51, 1.30 | 0.865  0.535  0.591  0.086  0.392 | 0.347 |
| CM’s occupation (*ref: Professional)*  Intermediate  Skilled  Partly skilled  Unskilled  Others | 0.97  0.87  0.88  1.12  0.99 | 0.73, 1.28  0.65, 1.16  0.63, 1.23  0.72, 1.75  0.72, 1.36 | 0.807  0.347  0.475  0.611  0.965 | 0.456 |
| CM’s education *(ref: NVQ level 5)*  No qualification  NVQ level 1  NVQ level 2  NVQ level 3  NVQ level 4 | 0.98  1.01  0.96  0.76  0.80 | 0.73, 1.32  0.75, 1.36  0.74, 1.23  0.58, 1.00  0.62, 1.03 | 0.895  0.960  0.731  0.053  0.086 | 0.046 |
| Housing tenure *(ref: Others)*  Owner-occupier | 1.05 | 0.91, 1.22 | 0.505 | 0.505 |

*n=number of observations; OR = odds ratio; CI = confidence intervals*

*Overall p-values for independent categorical variables with more than two values or categories were obtained using a post-estimation test, “mi test” command.*

******The p-value remained statistically significant*** *after correcting for false discovery rates using Benjamini-Hochberg (BH) method (see Table S7).*

*Associations between sociodemographic characteristics and the outcome were fitted in the same regression model, controlling for baseline BMI and the cohort.*

**Table S6.** Psychological well-being related factors and transitioning from overweight to normal weight (using pooled analytical sample sizes from both cohorts)

| **Psychological well-being related factors** | **Transitioning from overweight to normal weight vs. persistence** | | | |
| --- | --- | --- | --- | --- |
|  | **n** | **OR** | **95% CI** | **p-value** |
| Depressive symptoms | 14,209 | 1.03 | 0.97, 1.10 | 0.341 |
| Life satisfaction | 12,984 | 1.03 | 0.96, 1.10 | 0.475 |
| Self-efficacy | 11,817 | 1.03 | 0.95, 1.11 | 0.497 |

*n=number of observations; OR = odds ratio; CI = confidence intervals*

*Depressive symptoms, life satisfaction, and self-efficacy were transformed into z-scores.*

*Associations between psychological well-being related factors and the outcome were fitted in separate regression models, controlling for sociodemographic covariates, baseline BMI, and the cohort.*

**Table S7.** Adjustments for p-values from additional analyses using the Benjamini-Hochberg (BH) method

| **Tables** | **Cohort studies - Independent - dependent variables** | **p-value** | **Rank** | **(i/n)q** |
| --- | --- | --- | --- | --- |
| S5 | NCDS&BCS - Age | <0.001 | 1 | 0.001 |
| S5 | NCDS&BCS - Sex | **<0.001** | **2** | **0.002** |
| S3 | NCDS&BCS - Depressive symptoms - Changes in BMI | 0.028 | 3 | 0.003 |
| S5 | NCDS&BCS - Education | 0.046 | 4 | 0.004 |
| S4 | NCDS&BCS - Depressive symptoms - Changes in BMI | 0.062 | 5 | 0.005 |
| 5 | BCS - Depressive symptoms - Changes in BMI | 0.067 | 6 | 0.006 |
| 4 | BCS - Depressive symptoms - Transition to normal weight | 0.100 | 7 | 0.007 |
| S1 | NCDS&BCS - Life satisfaction - Changes in BMI | 0.105 | 8 | 0.008 |
| 5 | NCDS - Depressive symptoms - Changes in BMI | 0.106 | 9 | 0.008 |
| 3 | NCDS - Life satisfaction - Changes in BMI | 0.118 | 10 | 0.009 |
| S2 | NCDS&BCS - Self-efficacy - Transition to normal weight | 0.138 | 11 | 0.010 |
| S2 | NCDS&BCS - Depressive symptoms - Transition to normal weight | 0.153 | 12 | 0.011 |
| S4 | NCDS&BCS - Depressive symptoms - Transition to normal weight | 0.156 | 13 | 0.012 |
| S1 | NCDS&BCS - Self-efficacy - Changes in BMI | 0.157 | 14 | 0.013 |
| 3 | BCS - Self-efficacy - Changes in BMI | 0.201 | 15 | 0.014 |
| 3 | BCS - Depressive symptoms - Changes in BMI | 0.205 | 16 | 0.015 |
| S4 | NCDS&BCS - Life satisfaction - Changes in BMI | 0.238 | 17 | 0.016 |
| S3 | NCDS&BCS - Depressive symptoms - Transition to normal weight | 0.243 | 18 | 0.017 |
| 5 | NCDS - Self-efficacy - Changes in BMI | 0.259 | 19 | 0.018 |
| S1 | NCDS&BCS - Depressive symptoms - Transition to normal weight | 0.278 | 20 | 0.019 |
| 5 | BCS - Life satisfaction - Changes in BMI | 0.317 | 21 | 0.020 |
| 4 | BCS - Life satisfaction - Transition to normal weight | 0.327 | 22 | 0.021 |
| S4 | NCDS&BCS - Self-efficacy - Transition to normal weight | 0.339 | 23 | 0.022 |
| S6 | NCDS&BCS - Depressive symptoms - Transition to normal weight | 0.341 | 24 | 0.023 |
| S5 | NCDS&BCS - Father's occupation | 0.347 | 25 | 0.024 |
| 3 | BCS - Life satisfaction - Changes in BMI | 0.357 | 26 | 0.025 |
| S5 | NCDS&BCS - Ethnicity | 0.371 | 27 | 0.025 |
| 4 | BCS - Self-efficacy - Transition to normal weight | 0.384 | 28 | 0.026 |
| 3 | NCDS - Self-efficacy - Changes in BMI | 0.404 | 29 | 0.027 |
| S1 | NCDS&BCS - Depressive symptoms - Changes in BMI | 0.418 | 30 | 0.028 |
| S3 | NCDS&BCS - Life satisfaction - Changes in BMI | 0.429 | 31 | 0.029 |
| S3 | NCDS&BCS - Life satisfaction - Transition to normal weight | 0.440 | 32 | 0.030 |
| S5 | NCDS&BCS - Occupation | 0.456 | 33 | 0.031 |
| S6 | NCDS&BCS - Life satisfaction - Transition to normal weight | 0.475 | 34 | 0.032 |
| S4 | NCDS&BCS - Life satisfaction - Transition to normal weight | 0.494 | 35 | 0.033 |
| S6 | NCDS&BCS - Self-efficacy - Transition to normal weight | 0.497 | 36 | 0.034 |
| S5 | NCDS&BCS - Housing tenure | 0.505 | 37 | 0.035 |
| S2 | NCDS&BCS - Life satisfaction - Changes in BMI | 0.548 | 38 | 0.036 |
| S1 | NCDS&BCS - Life satisfaction - Transition to normal weight | 0.558 | 39 | 0.037 |
| S2 | NCDS&BCS - Life satisfaction - Transition to normal weight | 0.598 | 40 | 0.038 |
| S3 | NCDS&BCS - Self-efficacy - Changes in BMI | 0.618 | 41 | 0.039 |
| S1 | NCDS&BCS - Self-efficacy - Transition to normal weight | 0.672 | 42 | 0.040 |
| 4 | NCDS - Self-efficacy - Transition to normal weight | 0.718 | 43 | 0.041 |
| 5 | BCS - Self-efficacy - Changes in BMI | 0.779 | 44 | 0.042 |
| S5 | NCDS&BCS - Marital status | 0.779 | 45 | 0.042 |
| S3 | NCDS&BCS - Self-efficacy - Transition to normal weight | 0.809 | 46 | 0.043 |
| 4 | NCDS - Depressive symptoms - Transition to normal weight | 0.825 | 47 | 0.044 |
| S2 | NCDS&BCS - Depressive symptoms - Changes in BMI | 0.832 | 48 | 0.045 |
| S4 | NCDS&BCS - Self-efficacy - Changes in BMI | 0.870 | 49 | 0.046 |
| S2 | NCDS&BCS - Self-efficacy - Changes in BMI | 0.891 | 50 | 0.047 |
| 3 | NCDS - Depressive symptoms - Changes in BMI | 0.919 | 51 | 0.048 |
| 4 | NCDS - Life satisfaction - Transition to normal weight | 0.922 | 52 | 0.049 |
| 5 | NCDS - Life satisfaction - Changes in BMI | 0.965 | 53 | 0.050 |
| *i = the individual p-value’s rank; n = total number of tests (53); q = the false discovery rate (5%)* | | | | |
| ***The largest p-value lower than the Benjamini-Hochberg critical value is <0.001 (Rank 2). This p-value and all smaller p-values will be considered statistically significant.*** | | | | |
